# Good stability of a cementless, anatomically designed femoral stem in aging women: a 9-year RSA study of 32 patients

**DOI:** 10.1080/17453674.2018.1490985

**Published:** 2018-07-10

**Authors:** Erik Aro, Jessica J Alm, Niko Moritz, Kimmo Mattila, Hannu T Aro

**Affiliations:** a Department of Orthopaedic Surgery and Traumatology, Turku University Hospital and University of Turku, Turku;; b Department of Diagnostic Imaging, Turku University Hospital, Turku, Finland

## Abstract

Background and purpose — We previously reported a transient, bone mineral density (BMD)-dependent early migration of anatomically designed hydroxyapatite-coated femoral stems with ceramic–ceramic bearing surfaces (ABG-II) in aging osteoarthritic women undergoing cementless total hip arthroplasty. To evaluate the clinical significance of the finding, we performed a follow-up study for repeated radiostereometric analysis (RSA) 9 years after surgery.

Patients and methods — Of the 53 female patients examined at 2 years post-surgery in the original study, 32 were able to undergo repeated RSA of femoral stem migration at a median of 9 years (7.8–9.3) after surgery. Standard hip radiographs were obtained, and the subjects completed the Harris Hip Score and Western Ontario and McMaster Universities Osteoarthritis Index outcome questionnaires.

Results — Paired comparisons revealed no statistically significant migration of the femoral stems between 2 and 9 years post-surgery. 1 patient exhibited minor but progressive RSA stem migration. All radiographs exhibited uniform stem osseointegration. No stem was revised for mechanical loosening. The clinical outcome scores were similar between 2 and 9 years post-surgery.

Interpretation — Despite the BMD-related early migration observed during the first 3 postoperative months, the anatomically designed femoral stems in aging women are osseointegrated, as evaluated by RSA and radiographs, and exhibit good clinical function at 9 years.

The femoral component of the ABG (Anatomique Benoist Girard) hip prosthesis with mirrored geometries for left and right hips was designed to accommodate the natural anatomy of the proximal femur. The 3 goals were to create postoperative stability, ensure osseointegration of the proximal part of the stem coated with hydroxyapatite (HA), and promote proximal load transfer to prevent stress shielding (Van Rietbergen and Huiskes 2001, Van Der Wal et al. 2008). Surgical preparation, consisting of distal reaming and proximal broaching, is less forgiving with anatomic stem designs (Khanuja et al. 2011, Giebaly et al. 2016). Although the ABG-II stem is prone to periprosthetic fractures in the early postoperative period (Mäkelä et al. 2010, Thien et al. 2014, Catanach et al. 2015), it has demonstrated a high survival rate in prospective studies (Epinette et al. 2013, Herrera et al. 2013) and received acceptance in the Dutch national evaluation based on a 10-year survival of 91–93% in the Australian implant register (Poolman et al. 2015). A similar 10-year survival rate is noted in the Finnish Arthroplasty Register (2018).

Considering the original design concepts, it is not surprising if the clinical performance of the ABG-II stem deviates in postmenopausal women due to the mismatch with the proximal femoral endosteal geometry. The proximal femur of postmenopausal women frequently undergoes age-related widening of the intramedullary canal (Noble et al. 1995, Casper et al. 2012) and endosteal trabeculation of the cortical bone (Zebaze et al. 2010) with a frequent (> 60–70%) presence of low bone mineral density (BMD) (Glowacki et al. 2003, Mäkinen et al. 2007). Indeed, we observed an increased migration of ABG-II stems in female patients with low BMD during the first 3 months but not thereafter (Aro et al. 2012). In logistic regression analyses, signs of continued migration related to BMD, age, and canal flare index were also noted. In response to these observations, we invited the same cohort for the re-evaluation of stem stability. The participants underwent repeated radiostereometric analysis (RSA) of stem migration during the 2- and 9-year postoperative periods.

## Patients and methods

### Study design

This is a follow-up study of the 2-year single-center RSA study on female patients with hip osteoarthritis (Aro et al. 2012). The subjects underwent a preoperative screening of skeletal status (Mäkinen et al. 2007). The study protocol, inclusion and exclusion criteria, and the screening process have been reported previously (Aro et al. 2012). Patients underwent cementless total hip arthroplasty at the Turku University Hospital between August 2003 and March 2005. The surgery was performed using an anterolateral Hardinge approach. All patients received a custom-modified CE-certified ABG-II hip prosthesis with an anatomically designed hydroxyapatite-coated femoral stem with a non-modular neck, a hydroxyapatite-coated hemispherical acetabular cup, and ceramic-ceramic bearing surfaces (Stryker) (Aro et al. 2012, Finnilä et al. 2016). After surgery, patients were instructed to use crutches and partial weight-bearing up to 6 weeks.

**Figure 1.**
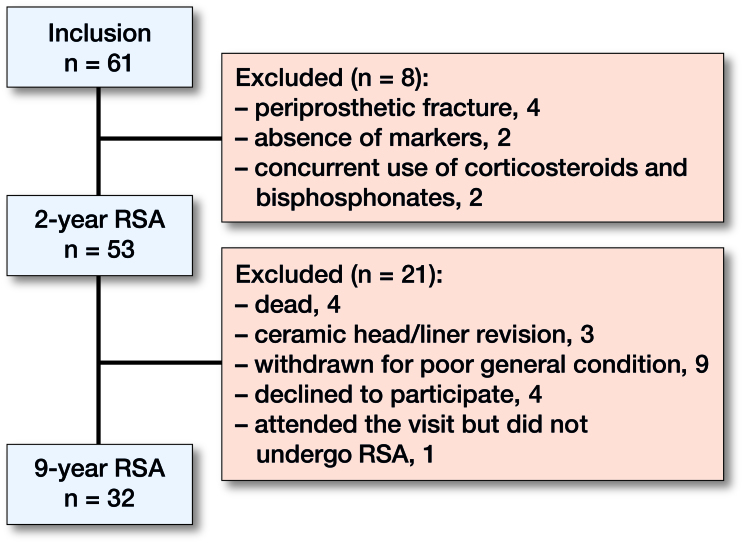
Patient flow throughout the study. Data from the first 2 years are presented in more detailed in a previous study (Aro et al. 2012).

The original cohort included 39 skeletally healthy patients with no history of osteoporosis treatment or any other medication affecting bone metabolism and a group of 14 patients with ongoing osteoporosis or corticosteroid treatment. The latter group did not differ in the 2-year RSA pattern of stem migration and were included in the current study. A cohort of 32 patients was able to attend the visit for repeated RSA imaging at 9 years (Table 1). The clinical outcome of the original cohort of 53 patients could be assessed (Figure 1). The 3 failure cases of ceramic-ceramic bearings have been discussed previously (Finnilä et al. 2016).

**Table 1.** Baseline demographics and clinical characteristics

No. of cases	32
Age, years	62 (41–78)
BMI (SD)	31 (6)
WHO classification of bone mineral density	
Normal bone (T-score ≥ –1.0	11
Osteopenia (–2.5 ≤ T-score < –1.0)	16
Osteoporosis (T-score < –2.5)	5
Dorr classification	
Type A	17
Type B	12
Type C	3
WOMAC score, (SD)	49 (16)
HHS score, (SD)	52 (15)

### RSA

**Figure 2.**
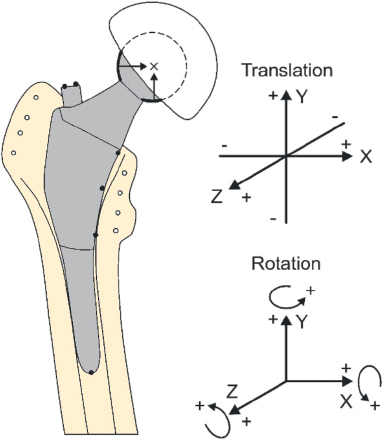
Schematic drawing of the prosthesis with 6 tantalum RSA stem markers and the coordinate system for RSA analysis of 3D micromotion of the femoral stem. In order to explicitly record the direction of micromotion, the directions are marked with + and – signs for both the translations and rotations.

The stem was marked by the manufacturer with 6 RSA beads (Figure 2). During surgery, multiple RSA markers (n = 4–7) were inserted into the greater and lesser trochanters. The center of the femoral head served as 1 additional marker. A standardized RSA technique was applied throughout the study. RSA imaging was performed using the uniplanar technique (Kärrholm et al. 1997), and image analysis was performed using UmRSA software (RSA Biomedical Innovations AB, Umeå, Sweden) according to RSA guidelines (Valstar et al. 2005). RSA was applied to access femoral stem micromotions along and around 3 axes (x, y, z) (Figure 2) and comparison was made with the baseline position. Since the translations along the x-axis and z-axis are difficult to interpret, the focus was on the translation along the y-axis and rotations around the 3 axes. Stability and adequate distribution of the markers were assessed by calculating the mean error of rigid body fitting (ME) and the condition number (CN) (Valstar et al. 2005). At 9 years, the mean ME was 0.27, and the mean CN was 71. The precision of the measurements was validated in each patient via double examination. As recommended (Ranstam et al. 2000, Derbyshire et al. 2009), the precision for each axis (Table 2) was calculated as standard deviations multiplied by the Student’s t distribution with n degrees of freedom for the mean value of differences between the double examinations.

**Table 2.** Precision of radiostereometric analysis based on double examinations

	Translation (mm)			Rotation (°)		
	x-axis	y-axis	z-axis	x-axis	y-axis	z-axis
Clinical precision	0.13	0.22	0.36	0.53	1.95	0.19

Clinical precision =2.05 x standard deviation for the mean difference between the double examinations.

### Clinical outcome

Standard 2-plane hip radiographs were obtained at 2 and 9 years. Radiographic signs of osseointegration were evaluated and classified according to Engh et al. (1990). Hip function was evaluated using the Harris Hip Score (HHS) and Western Ontario and McMaster Universities Osteoarthritis Index (WOMAC) (SooHoo et al. 2007).

### Sample size and statistics

Four patients were excluded from the RSA analysis due to inconsistent time-related values of translation or rotation related to either high ME (range 0.53–2.30) or high CN (185). Of the excluded patients, 2 were characterized by exceptionally high BMI (38–48). Thus, the RSA analysis included 28 patients out of 32 fulfilling the requirement for the minimum number of subjects (15–25 per group) (Valstar et al. 2005). Due to the limited group size, no subgroup analysis of patients with normal or low BMD was performed. HHS and WOMAC data were normally distributed and the significance of differences between 2 and 9 years were tested by paired t-test. Nonparametric tests were applied in the statistical analysis of RSA data. Data analysis was performed using SPSS software (IBM SPSS version 25.0; IBM Corp, Armonk, NY, USA). P-values <0.05 were considered significant.

### Ethics, funding, and potential conflicts of interests

The study extension was approved by the Ethics Committee of the Hospital District of Southwest Finland. Informed consent was obtained from all patients. The current study and the original study were supported financially by Competitive Research Funding of the Hospital District of Southwest Finland. The original study was also financially supported by the Academy of Finland and Stryker Inc., which took no part in the organization of the study, in analysis of the results, or writing of the manuscript. No competing interests were declared.

## Results

**Figure 3.**
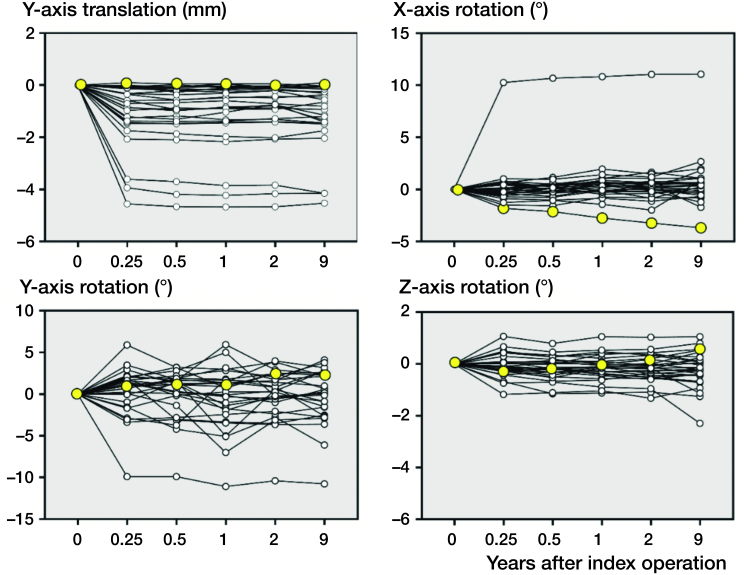
The migration pattern of individual femoral stems (n = 28) during the 9-year follow-up. 1 patient exhibited continuous x-axis rotation (yellow-filled markers).

Based on the measurements of translation and rotation on the 3 axes, RSA exhibited no statistically significant femoral stem migration between 2 and 9 years after surgery (Table 3). The low precision of the y-axis rotation (Table 2) caused intra-individual variation of the measured internal rotation around the y-axis (Figure 3). As a comparison, the high precision of the y-axis translation (subsidence) was associated with constant values (Figure 3).

**Table 3.** Femoral stem migration between 2 and 9 years

	2 years median (range)	9 years**^a^**median (range)	Difference (95% CI of median)	p-value**^b^**
Translation compared with baseline, mm				
x-axis	0.02 (–0.46 to 2.58)	0.00 (–0.48 to 2.80)	–0.02 (–0.08 to 0.07)	0.7
y-axis	–0.73 (–4.67 to 0.05)	–0.73 (–4.53 to 0.11)	–0.05 (–0.08 to 0.03)	0.2
z-axis	–0.25 (–1.03 to 2.17)	–0.29 (–1.79 to 1.52)	–0.07 (–0.17 to 0.13)	0.3
Rotation compared with baseline, degrees				
x-axis	0.18 (–3.23 to 11.04)	0.39 (–3.68 to 11.06)	0.02 (–0.14 to 0.18)	0.6
y-axis	0.34 (–10.38 to 4.00)	0.24 (–10.76 to 4.12)	0.09 (–0.69 to 0.73)	0.9
z-axis	–0.16 (–1.32 to 1.03)	–0.21 (–2.29 to 1.05)	0.02 (–0.17 to 0.12)	1.0

**^a^**Range of follow-up 7.8–9.3 years.

**^b^**Related-samples Wilcoxon signed rank test.

After the cessation of early migration during the first 3 postoperative months, 1 patient exhibited progressive, albeit minor, posterior tilt (rotation around the x-axis) (Figure 3, data points marked in yellow). Migration was 0.5° between 2 years and 9 years, close to the RSA precision for the rotation around the x-axis.

Patient-reported outcome scores exhibited no statistically significant changes between 2 and 9 years. The mean HHS of the cohort (n = 32) was 87 (95% CI 81–91) at 2 years and 85 (CI 78–91) at 9 years. The mean WOMAC was 15 (CI 11–20) at 2 years and 19 (CI 12–26) at 9 years.

All of the stems were classified as stable (overall score >10) according to the mean fixation and stability score (Engh et al. 1990). The average score of the cohort (n = 32) was 21 (18–27). The patient with suspected stem migration (Figure 3) did not show radiographic signs of implant loosening and exhibited a high (21) mean fixation and stability score. There was no radiolucency at the bone–implant interference, and all stems exhibited endosteal bone bridging (“spot welds”) as a sign of osseointegration. The spot welds were constantly located at the cortico-metaphyseal junction corresponding to the border between the HA-coated proximal stem and the polished distal stem. During the 9-year follow-up of the cohort, none of the stems were revised for loosening or infection.

To explore the clinical significance, the patient with suspected continuous stem migration was followed for an additional 5 years based on the review of electronic chart records. During the follow-up including hip radiographs at 14 years, the patient did not develop stem loosening.

## Discussion

Our previous results (Aro et al. 2012) questioned the early stability of uncemented ABG-II stems in aging women, but the current study revealed good stability at 9 years postoperatively. Based on the clinical and radiographic evaluations and RSA performed at 9 years, apart from 1 possible exception, ABG-II stems were “well-fixed without migration,” fulfilling the classical mechanical definition of osseointegration as discussed by Ryd (2006). ABG-II stems also exhibited “definite roentgenographic signs of bone ingrowth” (Engh et al. 1990). Thus, the stems appeared to have successfully osseointegrated during the first 2 years. In 1 patient, we could not exclude the possibility of stem loosening, which typically represents a continuous migration pattern over time (Kärrholm 2012). However, the extended follow-up did not demonstrate any clinical consequence. Thus, failed osseointegration or late loosening seem to be unlikely.

The critical question relates to the upper limit and time frame for acceptable early migration of uncemented femoral stems. As stated previously (Kärrholm 2012), the stems should preferably not migrate at all. However, many designs migrate, and the maximum time limit for subsidence appears to be 1 year. Consistent with the results from previous 5-year (Wolf et al. 2010, Weber et al. 2014) and 6-year (Callary et al. 2012) studies on non-anatomical femoral stem designs, the current study suggests that limited early migration is not harmful. An uncemented femoral component designed specifically for total hip arthroplasty of elderly hip fracture patients has demonstrated pronounced early migration in patients with low periprosthetic BMD, but no component migrated after 3 months (Sköldenberg et al. 2011). In contrast to previous studies of ABG-I prostheses in male and female patients less than 65 years of age (Thien et al. 2007, Nysted et al. 2014), our study focused exclusively on aging women who suffer more frequently from age-related anatomic abnormalities of the proximal femur compared with men (Noble et al. 1995). Our original analysis demonstrated that preoperative BMD dictates subsidence (translation along the y-axis) to a certain extent during the first 3 months after surgery (Aro et al. 2012). The measured subsidence in patients with normal BMD (mean 0.5 mm) (SD 0.5) and low BMD (1.1 mm) (SD 0.9) seemed to be within acceptable ranges because the clinical outcome scores of the patients with normal and low BMD were similar at 2 years, and the current analysis suggested uniform clinical recovery and implant healing. Notably, the RSA-measured subsidence differs from the clinical term of stem subsidence, which is evident on plain radiographs and carries a natural risk of impaired functional outcome. In our cohort, 3 patients exhibited excessive stem subsidence (> 3 mm) during the first 3–6 months (Figure 3). Interestingly, an analysis of potential risk factors for excessive subsidence may be more difficult than expected (White et al. 2012).

The histological analysis of retrieved ABG-II specimens has demonstrated an almost complete resorption of HA coating as a function of implantation time. The new bone incorporated with HA coating may be replaced by growing new bone (Tonino et al. 2009). We could not recognize any signs of late mechanical loosening of the stems. This finding is consistent with the reported acceptable 10-year survival of the stem (Epinette et al. 2013, Herrera et al. 2013) in addition to data from Australian and Finnish national registers. Interestingly, the stems constantly formed spot welds at the lower border of the HA-coated proximal stem. The site of spot welds corresponds to the region of the cortico-metaphyseal junction, which obviously transfers the load from the prosthesis to the cortical bone. This correlation is not precisely according to the original design (Van Rietbergen and Huiskes 2001, Van Der Wal et al. 2008), which aimed to achieve full loading of the HA-coated region of the stem.

As a limitation, not all subjects participated in the repeated examination at 9 years. However, we were able to track the clinical outcomes of all 53 participants of the original cohort, and none of the stems were revised for mechanical loosening or infection. In this respect, the study group of the current 9-year evaluation was not biased. Our study was also weak in the detection of the main stem rotation around the y-axis. We paid attention to the quality of our RSA imaging setup both for marker-based and markerless RSA of cementless femoral stems (Mäkinen et al. 2004, Nazari-Farsani et al. 2016). In our hands, the precision for measurement of y-axis rotation has been at the same level as the marker-based RSA (0.71°) and model-based RSA (0.79 degrees). These values are higher or approximately at the same level as the precision values reported in recent clinical trials (Li et al. 2014, Weber et al. 2014). Collaborating with our previous analysis of the ABG-II stem (Aro et al. 2012), the precision for the measurement of y-axis rotation was suboptimal (1.95 degrees). This finding could be explained by the fact that the femoral head was poorly visible due to the use of ceramic-ceramic articulation. The visibility of the RSA markers on the medial surface of the stem was not good either. The analysis would also benefit from the implantation of an increased number of bone markers. The number of markers is one of the critical determinants of RSA precision (Kärrholm et al. 1997). In a patient with a low number (4 or 5) bone markers, the loss of even 1 marker, for example due to late bone resorption of the calcar region, may jeopardize the spatial contribution. The analysis of individual migration patterns demonstrated the consequences caused by the imprecision. The data on stem rotation around the y-axis suffered from the noise caused by the low precision throughout the follow-up visits.

In conclusion, the anatomically designed uncemented femoral stem, which exhibited BMD-dependent early migration in aging women, currently exhibits signs of osseointegration, as evaluated by RSA and radiographic images, and acceptable clinical outcome scores in our 9-year prospective extension study.

EA collected data and wrote the manuscript, JJA collected data, NM supervised RSA, KM supervised radiographic imaging, and HTA initiated the study, operated on patients, and wrote the manuscript. All the authors approved the final manuscript.

The authors would like to thank Satu Honkala, RN, for the study coordination.

*Acta* thanks Johan Kärrholm and Stephan Maximilian Röhrl for help with peer review of this study.
